# Drawing Comparisons between SARS-CoV-2 and the Animal Coronaviruses

**DOI:** 10.3390/microorganisms8111840

**Published:** 2020-11-23

**Authors:** Souvik Ghosh, Yashpal S. Malik

**Affiliations:** 1 Department of Biomedical Sciences, Ross University School of Veterinary Medicine, Basseterre 334, Saint Kitts and Nevis; 2 College of Animal Biotechnology, Guru Angad Dev Veterinary and Animal Science University, Ludhiana 141004, India; malikyps@gmail.com

**Keywords:** coronavirus, SARS-CoV-2, animal coronaviruses, evolution, transmission, pathogenesis, therapeutics, prophylaxis

## Abstract

The COVID-19 pandemic, caused by a novel zoonotic coronavirus (CoV), SARS-CoV-2, has infected 46,182 million people, resulting in 1,197,026 deaths (as of 1 November 2020), with devastating and far-reaching impacts on economies and societies worldwide. The complex origin, extended human-to-human transmission, pathogenesis, host immune responses, and various clinical presentations of SARS-CoV-2 have presented serious challenges in understanding and combating the pandemic situation. Human CoVs gained attention only after the SARS-CoV outbreak of 2002–2003. On the other hand, animal CoVs have been studied extensively for many decades, providing a plethora of important information on their genetic diversity, transmission, tissue tropism and pathology, host immunity, and therapeutic and prophylactic strategies, some of which have striking resemblance to those seen with SARS-CoV-2. Moreover, the evolution of human CoVs, including SARS-CoV-2, is intermingled with those of animal CoVs. In this comprehensive review, attempts have been made to compare the current knowledge on evolution, transmission, pathogenesis, immunopathology, therapeutics, and prophylaxis of SARS-CoV-2 with those of various animal CoVs. Information on animal CoVs might enhance our understanding of SARS-CoV-2, and accordingly, benefit the development of effective control and prevention strategies against COVID-19.

## 1. Introduction

Since its first detection in cases with atypical pneumonia from the city of Wuhan, China, in December of 2019, the novel coronavirus (CoV), designated as SARS-CoV-2, has infected 46,182 million people, resulting in 1,197,026 deaths worldwide (as of 1 November 2020) [1,2,3,4]. The ensuing pandemic, known as COVID-19, is one of unprecedented proportions that has crippled the global economy and exerted far-reaching, detrimental impacts on societies [5,6]. The complex origin, extended human-to-human transmission, pathogenesis including immunopathology, and the various clinical presentations of SARS-CoV-2 have presented serious challenges to the scientific and medical communities towards properly understanding and combating the pandemic situation [3,7,8,9,10,11]. Data available so far strongly indicated that SARS-CoV-2 is a zoonotic virus that might have been derived from bats with, or without, the involvement of another animal species (pangolins?) as intermediate host/s [11]. SARS-CoV-2 is the 7th CoV to be reported in humans, and with respect to novelty, differs from the other important human respiratory CoVs: SARS-CoV and MERS-CoV [2,3,11,12].

Animal CoVs have been known to cause important diseases in livestock and companion animals [13,14,15,16,17,18,19,20,21,22,23]. Although human CoVs received attention only after the SARS-CoV outbreak of 2002–2003 [24], animal CoVs have been studied extensively for many decades, providing a plethora of important information on their complex genetic diversity, transmission, tissue tropism and pathology, host immune responses, therapeutics, and prophylaxis, some of which have striking resemblance to those seen with SARS-CoV-2. Moreover, the evolution of human CoVs including SARS-CoV-2 is intermingled with those of animal CoVs. Information on animal CoVs might enhance our understanding of SARS-CoV-2, and accordingly, benefit the development of effective control and prevention strategies against COVID-19. Since January 2020, many review articles have been written on the COVID-19 pandemic. However, these were mostly focused on various aspects of SARS-CoV-2 in humans. In this comprehensive review, attempts have been made to compare the current knowledge on evolution, transmission, pathogenesis, therapeutics, and prophylaxis of SARS-CoV-2 with those of CoVs in various animals.

## 2. Taxonomy of Coronaviruses

**Figure 1:**
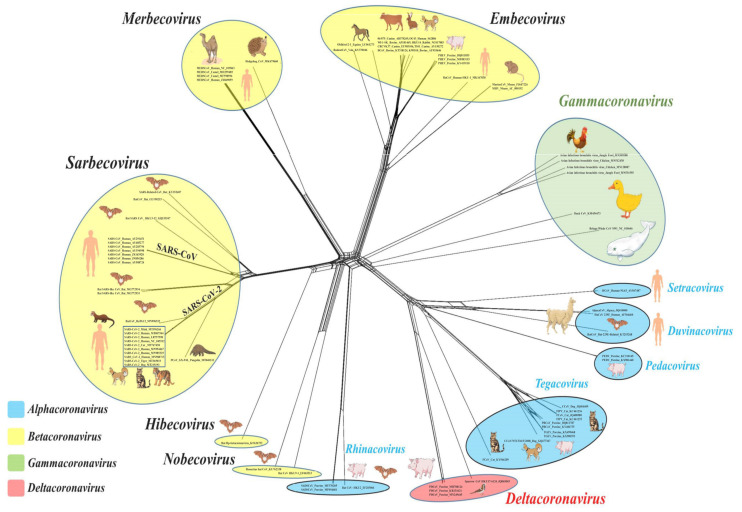
Neighbor-net phylogenetic grouping of representative animal and human coronaviruses (CoVs). Blue, yellow, green, and rose circles indicate the genus *Alphacoronavirus* (subgenera *Duvinacovirus*, *Pedacovirus*, *Rhinacovirus*, *Setracovirus*, and *Tegacovirus*), *Betacoronavirus* (subgenera *Embecovirus*, *Hibecovirus*, *Merbecovirus*, *Nobecovirus*, and *Sarbecovirus*), *Gammacoronavirus*, and *Deltacoronavirus*, respectively. Full-length sequences (*n* = 78) of the spike protein encoding genes of human and animal CoVs were retrieved from the NCBI GenBank portal (https://www.ncbi.nlm. nih.gov/genbank/) and subjected to multiple alignment using the ClustalW program embedded in MEGA 7.0 software. The resultant alignment file was used to generate a neighbor-net phylogenetic tree with the SplitsTree 4.0 program (http://www.splitstree.org/).

The current International Committee on Taxonomy of Viruses (ICTV) classification system for CoVs is primarily based on comparative analysis of the replicative protein-encoding genes (3CLpro, NiRAN, RdRp, ZBD, and HEL1), assisted by the DivErsity pArtitioning by hieRarchical Clustering (DEmARC) software [25]. To date, at least 2 subfamilies, 5 genera, 28 subgenera, and 46 species have been recognized within the family *Coronaviridae* (order *Nidovirales*) [26]. Coronaviruses belong to the subfamily *Orthocoronavirinae* that consists of four genera: *Alphacoronavirus*, *Betacoronavirus*, *Gammacoronavirus*, and *Deltacoronavirus* [26]. The alphacoronaviruses and betacoronaviruses infect mammals and include important pathogens of livestock and companion animals (Figure 1, Table 1) [13,24]. On the other hand, the gammacoronaviruses and deltacoronaviruses have been mostly detected in avian species, although there are some reports from mammals, including detection of a gammacoronavirus in a beluga whale (Figure 1, Table 1) [13,24,27].

Among the human CoVs of zoonotic origin, SARS-CoV-2 and SARS-CoV have been assigned to the species *Severe acute respiratory syndrome-related coronavirus* within the subgenus *Sarbecovirus* (genus *betacoronavirus*) that also include the SARS-related CoVs (SARS-rCoVs) from bats and pangolins (Figure 1) [11,24,26]. Although only virus isolates from the 2002–2003 SARS-CoV-2 outbreak were confirmed to cause clinical SARS (severe acute respiratory syndrome) in humans, all members of *Sarbecovirus* have names derived from the SARS-CoV, which is a reflection of the phylogenetic grouping rather than clinical presentation of the virus [25]. Even though SARS-CoV-2 and SARS-CoV belong to the same species and bind to the same receptor (angiotensin converting enzyme II (ACE2)) on host cells, they share low sequence similarity (~79%) between themselves [11,28]. The MERS-CoVs from humans and dromedary camels were assigned to the species *Middle East respiratory syndrome-related coronavirus*, and together with the species *Pipistrellus bat coronavirus HKU5*, *Tylonycteris bat coronavirus HKU4*, and *Hedgehog coronavirus 1* constitute the subgenus *Merbecovirus* (Figure 1) [26]. The other two human betacoronaviruses, HCoV-OC43 (species *Betacoronavirus 1*) and HCoV-HKU1 (species *Human coronavirus HKU1*), are members of the subgenus *Embecovirus* that also includes CoVs detected in alpacas, bovines, dogs, dromedaries, pigs, and rodents (Figure 1) [13,26]. Human alphacoronaviruses HCoV-NL63 and HCoV-229E belong to the subgenera *Setracovirus* (also including species *NL63-related bat CoV strain BtKYNL63-9b*) and *Duvinacovirus*, respectively [26].

## 3. Coronavirus Morphology and Genome

**Figure 2:**
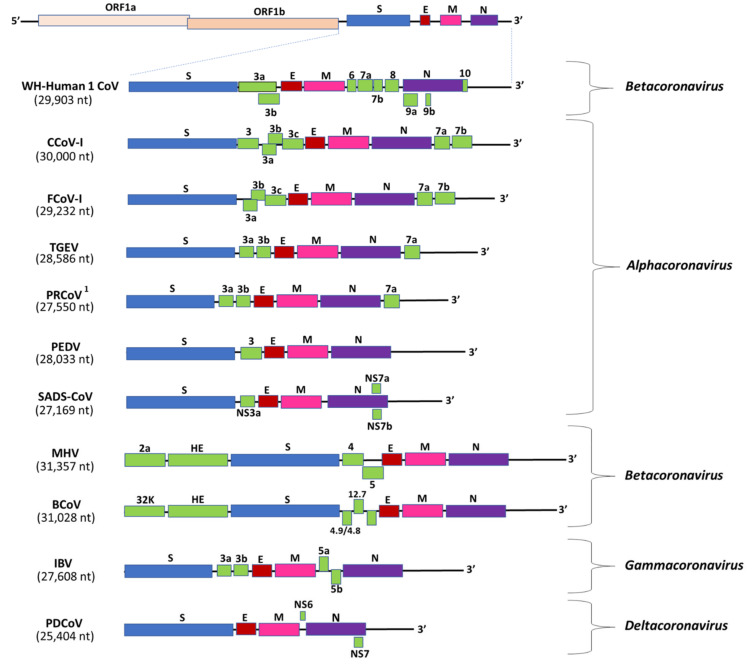
The genomic organization of SARS-CoV-2 (strain WH-Human 1 CoV) and representative animal coronaviruses (CoVs). The replicase gene constitutes two-thirds of the CoV genome, and consists of two overlapping open reading frames (ORFs), ORF 1a and 1b, which are translated into two large polypeptides, pp1a and pp1ab. The remaining 3′- region of the CoV genome (expanded in the figure) encodes the structural (spike (S), envelope (E), membrane (M), nucleocapsid (N)) and accessory proteins (highlighted with green boxes). The size of the complete genome of CoV is shown in parenthesis. ^1^ A large deletion (~600 nt at 5′- end) in the S gene transformed TGEV into PRCoV. Abbreviations: BCoV—bovine coronavirus; CCoV—canine coronavirus; CoV—coronavirus; FCoV—feline coronavirus; E—envelope; IBV—infectious bronchitis virus; M—membrane; MHV—mouse hepatitis virus; N—nucleocapsid; nt—nucleotide; ORF—open reading frame; PDCoV—porcine deltacoronavirus; PEDV—porcine epidemic diarrhea virus; PRCoV—porcine respiratory coronavirus; SADS-CoV—swine acute diarrhea syndrome coronavirus; S—spike; TGEV—transmissible gastroenteritis virus.

Coronaviruses are so called because of their crown-like morphology that resembles a solar corona in electron microscopy [43,44]. Virions are enveloped, spherical (sometimes pleomorphic), and ~80 to 120 nm in diameter [44]. Coronaviruses possess a positive sense, single-stranded RNA genome that is unusually large in size (~27 to 32 kb) compared to those of most other RNA viruses [45,46]. The CoV genome is organized into a 5′(capped)-leader-untranslated region (UTR) (~200-500 nucleotides (nt))-replicase-S (Spike)-E (Envelope)-M (Membrane)-N (Nucleocapsid)-UTR (~250–500 nt)-3′-poly (A) tail, and includes a variable number of accessory genes that are interspersed between the structural genes in the 3′- region of the viral genome [28,47] (Figure 2).

The replicase gene constitutes two-thirds of the CoV genome, and consists of two overlapping open reading frames (ORFs), ORF 1a and 1b, which are translated into two large polypeptides, pp1a and pp1ab, through a −1 frameshifting mechanism involving an RNA pseudoknot [15,47,48,49]. Eventually, both the polypeptides are proteolytically cleaved into 15, or 16 non-structural proteins (nsp). The CoV nsps form the replication–transcription complex (RTC) in double membrane vesicles, which drives the synthesis of genomic and subgenomic RNAs. The nested set of subgenomic RNAs, produced through discontinued transcription, serve as mRNAs for all the CoV structural and accessory genes.

The ORFs encoding the major CoV structural proteins (S, E, M, and N) are located in the one-third of the viral genome near the 3′-end (Figure 2) [15,47]. The S protein mediates virus attachment and fusion to the host cell membrane, which is crucial for determining tissue tropism and the host range of CoVs. The S protein is antigenically significant, as it is the primary inducer of host immune responses against CoVs [50,51,52]. The E protein is the smallest of the major structural proteins and has been proposed to play roles in viral replication, assembly, and pathogenesis [53]. However, for unknown reasons, the requirement of E protein has been shown to vary among CoVs. Murine CoVs produced viable replicating progeny in the absence of E protein, whilst lack of E arrested the trafficking and maturation of transmissible gastroenteritis virus (TGEV, a CoV in pigs), and generated attenuated SARS and propagation-defective MERS virus particles [54,55,56,57].

The M protein, with its three transmembrane domains, is the most abundant of the CoV structural proteins [49,58,59]. The M protein defines the shape of the viral envelope, regulates membrane curvature, and is considered as the central organizer of virus assembly [53,58,60]. The N protein complexes with the viral RNA to form a helical nucleocapsid, which is unusual for plus sense RNA viruses, and interacts with the nsp3 and M proteins to tether the viral genome to RTCs [47,49,61]. The N protein has also been proposed to play various other roles in virus replication, transcription, and translation, and modulate/inhibit host cellular responses, such as the inhibition of interferon production and viral suppressors of RNA silencing (VSR) [49,61,62]. Besides the four major structural proteins, members of the subgenus *Embecovirus* (genus *Betacoronavirus*) encode an additional structural protein, the hemagglutinin-esterase (HE), which is related to the HE fusion protein of Influenza C viruses (Figure 2) [63]. However, the HE of CoVs lacks membrane fusion activity [63].

The accessory genes are scattered among, or even overlap, the structural genes in the 3′- portion of the CoV genome, and can vary in number, size, and location between different CoVs (Figure 2) [64,65,66]. These genes do not exhibit significant homology among different subgroups of CoVs, and not much is known about the various functions of the proteins they encode. In general, the accessory genes/proteins are considered nonessential for virus replication in vitro. However, during in vivo infection, the CoV accessory proteins have been shown to play important roles in virus–host interactions [65,66,67,68,69]. Mouse hepatitis virus (MHV) exhibited reduced virulence in the absence of accessory proteins, whilst infectious bronchitis virus (IBV, a CoV of poultry) demonstrated enhanced virulence after mutation in the accessory (3b) gene [67,68]. Accessory proteins 3a and 8a were associated with compensatory functions in the absence of E protein in SARS-CoV. Accessory proteins have been involved in the modulation of cellular responses (programmed cell death, cytokine production, and interferon signaling) [65,69].

## 4. Evolution of Human and Animal Coronaviruses

**Figure 3:**
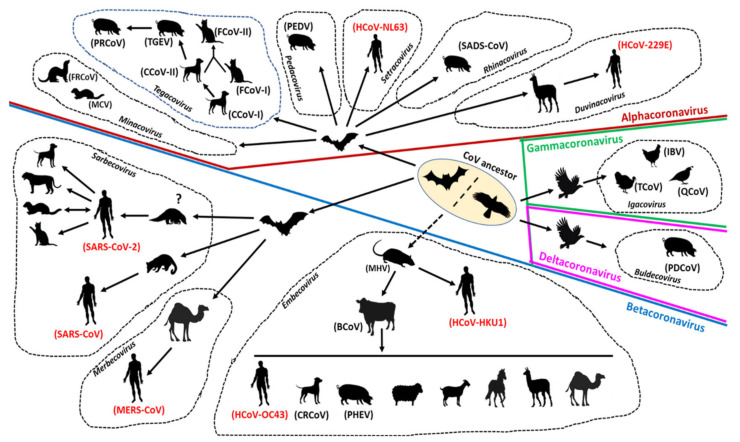
The evolution of animal and human coronaviruses (CoVs). The genera *alphacoronavirus*, *betacoronavirus*, *gammacoronavirus*, and *deltacoronavirus* have been delineated with dark red, blue, green, and pink lines, respectively. The subgenera of CoVs are demarcated with black dashed lines and indicated with italic type. The common names of CoVs are mentioned in parenthesis, and those of human CoVs are highlighted with red font. Abbreviations: BCoV—bovine coronavirus; CCoV—canine coronavirus; CRCoV—canine respiratory coronavirus; FCoV—feline coronavirus; FRCoV—ferret coronavirus; HCoV—human coronavirus; IBV—infectious bronchitis virus; MCV—mink coronavirus; MERS-CoV—middle east respiratory syndrome coronavirus; MHV—mouse hepatitis virus; PDCoV—porcine deltacoronavirus; PEDV—porcine epidemic diarrhea virus; PHEV—porcine hemagglutinating encephalomyelitis virus; PRCoV—porcine respiratory coronavirus; QCoV—quail coronavirus; SADS-CoV—swine acute diarrhea syndrome coronavirus; SARS-CoV—severe acute respiratory syndrome coronavirus; TCoV—turkey coronavirus; TGEV—transmissible gastroenteritis virus.

The evolution of CoVs is driven by mutation and recombination events, facilitated by the large size of the viral RNA genome that allows for extra plasticity, and has resulted in the emergence of CoVs with altered antigenicity, virulence, tissue tropism, and/or host range [13,24,70,71,72,73]. Although CoVs have genetic proofreading mechanisms [74,75], their estimated long term mutation rates (average of ~104 substitutions per year per site) are moderate-to-high compared to those of other single-stranded RNA (ssRNA) viruses [70], which, in part, may be attributed to the high replication rates of CoVs in host cells [11]. Because of their template switching ability during virus replication, CoVs are subjected to increasing rates of recombination [47,70,71]. Both homologous (between CoVs) and heterologous (with other viruses) recombination events have been documented for CoVs [63,70,76,77]. The evolution of CoVs in animals and humans is shown in Figure 3.

The possible roles of recombination and mutation in the origin and spread of SARS-CoV-2 have been evaluated [11,78]. The SARS-CoV-2 genome shared a maximum nucleotide sequence identity of 96.2% with that of bat CoV strain RaTG13 (from a horseshoe bat *(Rhinolophus affinis*) sampled in Yunnan province, China in 2013) [79]. However, the receptor binding domain (RBD) of SARS-CoV-2 shared only 85% similarity and differed in five of the six crucial amino acid residues with that of RaTG13 [11,78,79]. Interestingly, the RBD of SARS-rCoVs from Malayan pangolins (*Manis javanica*, illegally trafficked into animal markets in China) was closely related (97% amino acid sequence similarity and shared all the six critical amino acid residues shaping the ACE receptor) to that of SARS-CoV-2, although the rest of the pangolin CoV and SARS-CoV-2 genomes lacked significant sequence homology between each other [11,78,80]. Based on the above, it was speculated that the RBD region of the spike protein of SARS-CoV-2 may have been derived through recent recombination events in pangolins [78,80]. However, divergence time analysis of CoV coding sequences encompassing the critical amino acid sites did not corroborate the hypothesis [78]. Therefore, it might be possible the identical RBD sites in the SARS-CoV-2 and pangolin SARS-rCoV genomes resulted from coincidental convergent evolution [78,80]. The possibility of SARS-CoV-2 acquiring key mutations during cryptic transmission in humans prior to the first reported case in Wuhan in December 2019 has also been proposed [11]. Several nonsynonymous substitutions have been identified among SARS-CoV-2 strains circulating in human populations [7]. Notable among these are the D614G mutation in the spike protein gene that has been reported with increasing frequency in multiple countries [81], and a 382-nucleotide deletion in ORF8 of CoV genomes from a cluster of clinical cases in Singapore [82]. However, there is yet no conclusive evidence that these genetic changes influence the viral fitness, transmissibility, and/or pathogenesis of SARS-CoV-2 in humans [7,83].

Among the other important respiratory betacoronaviruses in humans, the progenitor of SARS-CoV was hypothesized to have originated from recombination events within bats, and eventually, was transmitted to masked palm civets (*Paguma larvata*) by feco–oral transmission [24,84,85]. Thereafter, the virus underwent rapid mutations in the S gene and orf8, and spread among market civets before infecting and adapting to humans. The MERS virus was speculated to have been introduced in dromedary camels from bats at least 30 years back, followed by spillover from camels to humans [24,48,86]. Comparisons of the genomes of bat MERS-rCoVs with those of camel and human MERS-CoVs suggested substantial evolution, including recombination events during the emergence of MERS-CoVs. In addition to betacoronaviruses, bat-derived alphacoronaviruses, HCoV-NL63, and HCoV-229E (through alpacas as intermediate host) have been detected in humans with mild respiratory infections [87,88]. Although intermediate animal hosts have been proposed in the zoonotic transmission of respiratory CoVs [11,24], (i) the detection of antibodies against the SARS-rCoV nucleocapsid in humans residing near a bat cave [89] and (ii) identification of all genetic elements required to form a SARS-CoV in a single bat cave [13,90] indicated the possibility of direct transmission events from bats to humans. Other than bats, rodents have been proposed as possible ancestors to human CoVs: HCoV-OC43 (through a bovine intermediate host) and HCoV-HKU1 (intermediate host, if any, unknown) [24,87,88].

Similar to the human CoVs, animal CoVs exhibit complex evolutionary pathways that reveal the genetic footprints of cross-species transmission events (Figure 3) [13,15]. By sequence comparisons and phylogenetic analyses, bats are believed to be the ancestors of alphacoronaviruses and betacoronaviruses, whilst rodents have been proposed to play important role/s in the evolution of members of the subgenus *Embecovirus* [13,91]. Furthermore, BCoVs, thought to be descendants of the rodent CoVs, were hypothesized to be involved in the evolution of non-bovine CoVs, such as canine respiratory CoVs (CRCoV), porcine hemagglutinating encephalomyelitis virus (PHEV), and equine CoVs [13,88,92]. On the other hand, birds are considered as reservoirs of the ancestors of gammacoronaviruses and deltacoronaviruses [13,93].

Recombination and mutation are intimately linked with the evolution of animal CoVs, and have been associated with changes in virulence, tissue tropism, and host specificity [13,14,15,17,18,21,91]. Canine CoV-II (CCoV-II) appears to have evolved from recombination events between CCoV-I and an unknown CCoV, resulting in the progressive loss of orf3 in the CCoV-II genome [15,94]. Furthermore, recombinant CCoV-II strains that are related to prototype TGEV-like strains in the 5′- and 3′- ends of the S gene, and classified as CCoV-IIb based on the amino acid sequence of the N-terminal region of the S protein, have been reported in Europe, Latin and Central America, China, and Southeast Asia [95,96,97,98,99]. Feline CoV-II (FCoV-II) appears to have originated through different recombination events involving FCoV-I and CCoV-II [15,17,100]. Although conclusive data are lacking, mutations (point mutations in S gene, or insertion/deletion in 3c, 7a, or 7b genes of FCoV) have been proposed to play role/s in the transformation of feline enteric CoV (FECoV, replicates in enterocytes of intestinal villi and causes mild diarrhea in cats) to the more virulent feline infectious peritonitis virus (FIPV, increased affinity for macrophages/monocytes and causes severe systemic disease and immunopathology) [13,15,17,101,102,103]. Similar to FCoVs, two distinct forms of CoV (ferret enteric CoV (FRECV) that causes epizootic catarrhal enteritis, and ferret systemic CoV (FRSCV) that causes a systemic disease that resembles FIPV) that are differentiated by low sequence identities in the S gene between FRECV and FRSCV and truncated orf3 product in FRSCV have been identified in ferrets [104,105]. Complete genomic analysis of ferret CoVs (FRCoVs) also revealed recombination events involving the S, 3c, and E genes [106,107].

Porcine CoVs are excellent examples of transmission of CoVs between unrelated host species. Based on the high genetic relatedness between CCoV-II and porcine transmissible gastroenteritis virus (TGEV), and the presence of remnants of orf3 in genomes of both CoVs, it has been proposed that TGEV originated from CCoV-II [18,19,20]. Although TGEV causes acute gastroenteritis in pigs, which can be fatal in piglets, a large deletion (~600 nt at 5′- end) in the S gene transformed TGEV into a mild porcine respiratory CoV, designated as PRCoV [19,20]. Porcine epidemic diarrhea virus (PEDV) was found to be genetically more closely related to bat CoV (BtCoV/512/2005) than those of other alphacoronaviruses including TGEV. Moreover, PEDV and bat CoVs shared signature motifs in the 5′-UTR, and PEDV has been shown to infect cells derived from bats [18,108]. Taken together, these observations suggested that PEDV might have originated from spillover of CoVs from bats to pigs [18,109]. PEDV genotypes (S-INDEL and NTD-del) with insertions/deletions in the S gene that cause mild clinical disease, and PEDV-TGEV recombinants have also been reported [18,108]. More recently, another porcine alphacoronavirus, swine enteric alphacoronavirus (SeACoV), also referred to as swine acute diarrhea syndrome coronavirus (SADS-CoV) or porcine enteric alphacoronavirus (PEAV), which emerged in Guangdong province in China in 2016, was derived from Rhinolophus bat CoV-HKU2-like strains [24,35,110]. The ancestor of porcine deltacoronavirus (PDCoV) was proposed to have originated through recombination events involving sparrow CoV HKU15 and bulbul CoV HKU11 [13,93].

Among avian CoVs (ACoVs), the evolution of IBV in poultry has been more studied compared to those of other viruses. High rates of mutation, especially in the hypervariable S1 domain, and inter-lineage recombination events including those between vaccine and wild strains, have resulted in the emergence of multiple IBV serotypes, genotypes, and pathotypes, some of which caused serious outbreaks and contributed to vaccine failure [21,22,39,111]. IBV was proposed to participate in recombination events with unknown ACoVs, resulting in the origin of turkey CoV (TCoV) and guinea fowl CoV (GfCoV) [112]. These events might have resulted in alterations in tissue tropism, as all IBV strains cause respiratory disease (although some enterotropic forms have also been detected), whilst TCoV and GfCoV are enteric pathogens [111,112]. Recombination events between IBV and TCoV have also been reported [113].

## 5. Transmission of SARS-CoV-2 and the Animal Coronaviruses

Since SARS-CoV-2 is predominantly regarded as a respiratory pathogen, direct contact and respiratory droplets have been considered as the primary routes of human-to-human transmission so far [8]. However, recent findings have raised speculations on the possible spread of SARS-CoV-2 by other routes, such as fecal–oral, airborne, contaminated surfaces, environmental wastewater, sexual, ocular, and vertical transmission [8,114]. Fecal–oral and airborne transmission routes of SARS-CoV-2 have received serious attention. The prolonged fecal shedding of the virus, especially in individuals with RT-qPCR negative throat swabs and/or without clinical signs, and detection of viral RNA in the pharynx, esophagus, gastric mucosa, rectal mucosa, and duodenal mucosa corroborate the fecal–oral transmission potential of SARS-CoV-2. Fecal–oral transmission has also been proposed to cause further spread by environmental contamination, posing serious risks to countries with poor sanitary conditions [114,115,116,117]. Although SARS-CoV-2 is not considered an airborne virus, adsorption of the virus on moisture laden dust/particulate matter could facilitate long distance transport, resulting in pollution-to-human transmission [118,119]. The viability of SARS-CoV-2 on various inanimate surfaces supports the prospects of indirect transmission through aerosol or contaminated surfaces/fomites [120].

Many of the animal CoVs (CCoV-I and -II, FCoV-I and -II, FRCoV, PEDV, PDCoV, TGEV, mink CoV (MCV), TCoV, quail CoV (QCoV), GfCoV) are enteric pathogens, and are mainly transmitted by the fecal–oral route [13,14,17,18,19,20]. Interestingly, enteric CoVs, such as PEDV and PDCoV, were shown to infect pigs via airborne transmission routes, possibly resulting from ingestion of aerosolized viral particles [121]. Respiratory animal CoVs, such as CRCoVs, are primarily transmitted via droplet infection, or direct contact with infected dogs, while porcine respiratory coronaviruses (PRCoVs) are derived from enteric CoV TGEV inside the porcine host [20,42,122]. Certain CoVs, such as bovine CoV (BCoV) in cattle and IBV of poultry, are shed in both feces and respiratory secretions [20,123,124]. Ocular transmission of BCoV, albeit in low titers, has been reported in a single study [125]. Although vertical transmission of CoVs in animals is not clearly established and is considered rare, a few studies have provided some preliminary evidence: (i) in utero transmission in mice following oronasal inoculation of MHV [126], (ii) detection of FCoV in newborn and still-born kittens born to a FIPV-positive queen cat [127], and (iii) the presence of IBV in day-old chicks and eggs from infected hens [124,128].

Differences in organotropism within the same CoV species have been shown to influence virus transmission routes, such as enterotropic MHV being shed in high titers in feces, whilst the respiratory MHV are transmitted via direct contact with infected mice [23]. Prolonged/recurrent shedding of virus/viral RNA without clinical signs, or subclinically from animals, has been reported with certain animal CoVs, mirroring similar findings with SARs-CoV-2; however, these shedding patterns might not always reflect the transmission potential of CoVs [17,18,19,20,21,124]. Indirect transmission of CoVs through contaminated surfaces/fomites/environment, feed/water, and/or people have been well documented in animals [14,17,18,19,20,21,22,42,123,124].

Reverse zoonosis of SARS-CoV-2 has been reported in companion animals (cats, dogs), captive wildlife (tigers, lions), and farmed minks (Figure 3) [29,129]. The virus was shown to be transmitted from infected to healthy cats under controlled conditions [130,131]. Studies based on experimental infections have shown that cats, ferrets, fruit bats, hamsters, nonhuman primates, tree shrews, and transgenic mice are susceptible, whilst dogs, pigs, and poultry are resistant to SARS-CoV-2 [29,129,132,133]. A recent review of the diversity and natural host range of betacoronaviruses in bats suggested that more than 40 species of temperate zone North American bats could be immunologically naive and susceptible to infection by SARS-CoV-2 [134]. On the other hand, a study in the Netherlands provided preliminary sequencing-based evidence for mink-to-human transmission of SARS-CoV-2 [135]. However, in another study, the virus was not detected in cats and dogs living in close contact with SARS-CoV-2-positive humans [136]. Although there is still a lack of solid evidence for natural animal-to-human transmission or sustained animal-to-animal transmission, these observations warrant further investigations of the transmission dynamics of SARS-CoV-2 between animals and humans [29]. Transmission of CoVs between different animal species has been discussed in the section on CoV evolution (Figure 3).

## 6. Pathogenesis of SARS-CoV-2 and the Animal Coronaviruses

The pathogenesis of SARS-CoV-2 in humans has been excellently reviewed by Tay et al. (2020) [137] and Gupta et al. (2020) [9]. SARS-CoV-2 appears to be less fatal, but far more infectious than MERS-CoV or SARS-CoV [2]. SARS-CoV-2 has been shown to cause substantial pulmonary disease, including pneumonia and acute respiratory distress syndrome (ARDS), and extrapulmonary clinical manifestations (cardiac, cutaneous, gastrointestinal, gustatory, hematological, hepatic, neurological, olfactory, ocular, and renal) have also been observed [9,138].

Following entry into the host, SARS-CoV-2 infects cells of the upper and lower respiratory tract [137]. Successful virus entry requires co-expression of ACE2 and TMPRSS2 (transmembrane protease, serine 2) on the surface of host cells. Although SARS-CoV-2 and SARS share the same host cell receptor (ACE2), SARS-CoV-2 RBD has more affinity for ACE2 than SARS-CoV, and the S protein of SARS-CoV-2 contains a furin-like cleavage site that is absent in SARS-CoV, which may, in part, explain the enhanced infectivity of SARS-CoV-2 over SARS-CoV. The receptor binding motif (RBM) of SARS-CoV-2 differs in five critical amino acid residues (Y455L, L486F, N493Q, D494S, and T501F) with that of SARS-CoV [139,140]. These residue changes have been proposed to stabilize two virus-binding hotspots at the SARS-CoV-2 RBD–ACE2 interface. Additionally, the RBM of SARS-CoV-2 contains a four-residue motif (amino acids 482–485, GVEG), which results in a more compact conformation of its ACE2-binding ridge compared to SARS-CoV, facilitating better contact with the N-terminal helix of ACE2 [139,140]. Increased atomic interactions have been observed between the C-terminal domain of SARS-CoV-2 S protein and human ACE2 compared to those between SARS-CoV RBD and human ACE2 [141]. Nanomechanical studies have revealed that the RBD of SARS-CoV-2 is more stable than that of SARS-CoV [142]. By combining sequence alignment, probability analysis, and binding free energy calculation, it has been hypothesized that a few residues on the viral RBM (452, 489, 500, 501, and 505) have high chances to mutate into more infectious SARS-CoV-2 strains [143]. On the other hand, non-RBD mutations have also been implicated to impact the transmissibility of the SARS-CoV-2, suggesting possible roles of non-RBD sites in influencing virus–receptor interactions [144]. The SARS-CoV-2 polybasic cleavage sites, although located ~10 nm away from the RBD, were shown to enhance the RBD−ACE2 binding affinity via electrostatic interactions and hydration [144]. The spike protein mutation D614G, which became dominant in the COVID-19 pandemic, was shown to enhance viral loads in the upper respiratory tract of experimentally inoculated hamsters, suggesting a possible role of the mutation in increased human-to-human transmission of SARS-CoV-2 [145].

**Figure 4:**
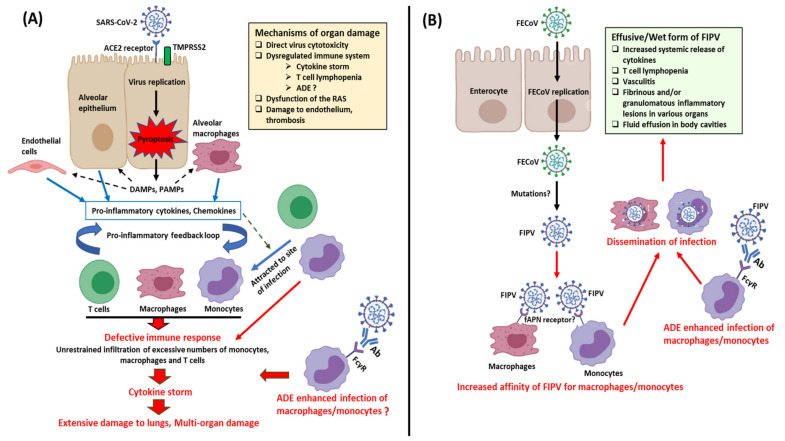
Mechanisms of immunopathogenesis induced by SARS-CoV-2 in humans (**A**), and feline coronavirus (CoV) in cats (**B**). The figure was created using icons from biorender.com (accessed 18 September 2020). Abbreviations: Ab—antibody; ACE2—angiotensin-converting enzyme 2; ADE—antibody-dependent enhancement; DAMPs—damage-associated molecular patterns; fAPN—feline aminopeptidase N; FcγR—Fc gamma receptor; FECoV—feline enteric coronavirus; FIPV—feline infectious peritonitis virus; PAMPs—pathogen-associated molecular patterns; RAS—renin–angiotensin system; TMPRSS2—transmembrane protease—serine 2.

Replication of SARS-CoV-2 induces pyroptosis of host cells, causing the release of damage-associated molecular patterns (DAMPs) (Figure 4). Neighboring epithelial cells, endothelial cells, and alveolar macrophages recognize the DAMPs as well as pathogen-associated molecular patterns (PAMPs), such as viral RNA from damaged cells, triggering increased secretion of pro-inflammatory cytokines and chemokines. Monocytes, macrophages, and T lymphocytes are eventually attracted to the site of infection, facilitating further inflammation and forming a pro-inflammatory feedback loop [137,146]. In a defective immune response, extensive lung damage ensues from increased accumulation of immune cells and overproduction of pro-inflammatory cytokines. The cytokine storm may spread to other organs, resulting in multi-organ damage (Figure 4). Other key mechanisms of multi-organ damage may include direct viral toxicity (corroborated by recovery of viral RNA from different organs), endothelial cell damage and thromboinflammation, and dysregulation of the renin–angiotensin system (RAS) [9]. Non-neutralizing anti-SARS-CoV-2 antibodies produced by B cells may exhibit antibody-dependent enhancement (ADE), promoting infection and aggravating organ damage [137]. T cell lymphopenia has been observed in cases with SARS-CoV-2 and may be due to the pulmonary recruitment of immune cells, direct virus killing of lymphocytes, and/or apoptosis of T cells [9].

Studies on the complex and evolving pathobiology of various animal CoVs have revealed the enigmatic nature of the virus. One of the best examples in this regard that also mirrors the immunopathology seen with SARS-CoV-2 is that of FCoV (Figure 4). Based on pathogenicity, FCoVs (both FCoV-I and FCoV-II) are classified into two biotypes, FECoV (causes mild diarrhea/asymptomatic) and FIPV (causes fatal systemic infection and immunodysregulation) [17]. According to the most accepted hypothesis, due to unknown reasons (possibly mutations), FECoV (primarily enterotropic) transforms into FIPV (replicate efficiently in monocytes/macrophages) in 5–10% of infected cats [103,147]. Virus infected monocytes/macrophages cause a systemic FIPV infection that is characterized by excessive systemic release of cytokines, depletion of lymphocytes, vasculitis, body cavity effusions, and fibrinous and/or granulomatous inflammatory lesions in various organs including the central nervous system (CNS) [16,17,103,148,149]. ADE of FIPV has been shown to enhance infection in cats, with implications on vaccine development [16,17]. Based on the host immune responses (cell-mediated immunity vs. humoral immunity), wet (effusive), dry (noneffusive), or mixed FIPV forms may be observed in cats [17,147]. Among the cytokines studied in association with FIPV, interleukin-6 (IL-6) exhibited the greatest hepatic upregulation, and showed significant activity in the more fatal effusive form (sera and ascitic fluids) compared to the dry form of the disease in cats [149,150]. Interestingly, elevated systemic levels of IL-6 have been associated with a poor prognosis of SARS-CoV-2 [151].

Similar to FCoVs, porcine CoVs have been found to switch tissue tropism within the host, as revealed by the transformation of TGEV (causes diarrhea) to PRCoV (infects upper and lower respiratory tract and causes mild respiratory disease) [18,20]. This alteration of tissue tropism has been attributed to a deletion in the 5′- end of the S gene of TGEV, which resulted in the loss of sialic acid binding activity (crucial for virus attachment to intestinal mucins and enteric infection) in PRCoV [152]. PRCoV infection in pigs confer partial immunity against TGEV, and therefore, widespread distribution of PRCoV may have accounted for a significant reduction in TGE outbreaks in swine herds worldwide [19,153]. Alteration of tissue tropism has also been observed for CCoVs. Although CCoVs (CCoV-I and CCoV-II) generally cause mild diarrhea or asymptomatic enteric infection, hypervirulent CCoV-IIa strains, designated as pantropic CCoVs, have been associated with systemic and fatal disease in dogs from Europe and South America since 2005 [14,99,154,155,156]. Pantropic CCoVs spread to extraintestinal tissues and are characterized by vomiting, hemorrhagic diarrhea, severe leukopenia, neurological signs (ataxia, seizures), severe lesions in the major organs, and death [14,99,154,155,156]. However, the molecular basis for enhanced virulence and expanded tissue tropism of pantropic CCoVs remains to be clearly elucidated [14,156]. Furthermore, experimental studies have revealed that the outcomes of pantropic CCoV infection may not be invariably fatal, and that the severe clinical signs are those from concurrent infections, aggravated by pantropic CCoV-induced leukopenia [14,157].

The involvement of multiple organs has been observed in the pathogenesis of respiratory CoVs in animals. BCoV infects the intestine and the upper and lower respiratory tract, and has been associated with three distinct clinical syndromes in cattle: calf diarrhea (1- to 3-week-old calves), winter dysentery (adult dairy and beef cattle), and calf respiratory disease (2- to 10-month-old cattle) [20,158,159]. Mild respiratory signs with concurrent diarrhea are usually seen in infected animals, which can be exacerbated by coinfections with other pathogens. Although the respiratory and enteric strains of BCoV are antigenically similar, they (including strains from the same animal) exhibit genetic differences (point mutations) between themselves. Furthermore, during cell culture, an enteric BCoV strain was found to accumulate mutations that resembled those of respiratory BCoV from the same animals [160]. Based on these observations, a quasispecies concept that confers certain viruses with enhanced ability to infect the respiratory tract than the gut, or vice versa, has been proposed [160]. The IBV of poultry has been subjected to rapid mutation rates, viral recombination, and host selection pressure, resulting in the emergence of strains with novel genotype, serotype, and/or pathogenicity including multiorgan tropisms [21,22,39,111,161,162]. Although the respiratory tract is the primary site of infection for all IBV strains (characterized by conjunctivitis, tracheitis, and ciliostasis), certain strains have been shown to cause renal (nephropathogenic: nephritis and urate deposition), reproductive (fall in egg production, misshaped eggs, and lesions in oviduct), and/or enteric disease. CRCoV (genus *betacoronavirus*) is predominantly a respiratory pathogen of dogs, and unrelated to the enteric CCoVs (genus *alphacoronavirus*) [14]. However, CRCoVs have been detected, albeit rarely, in extrapulmonary tissues (spleen, mesenteric lymph nodes, and intestines) of dogs including those with diarrhea, warranting further investigations on the tissue tropism of the virus [42,122]. Limited replication of PRCoVs have also been observed in the swine enterocytes [19,20].

The CNS is involved in the pathogenesis of MHV and PHEV. Two organotropic forms have been identified in MHVs: respiratory (polytropic) and enterotropic [23]. Following initial replication in the nasal respiratory and olfactory epithelium, the respiratory (polytropic) MHV strains spread into the brain, liver, lung, bone marrow, lymphoid tissue, and reproductive organs, whilst enterotropic MHV strains primarily replicate in the intestine, but can also spread to the liver, lymphoid tissue, and spleen [23]. MHVs have been associated with more severe disease in T cell-deficient compared to B cell-deficient, or immunocompetent mice, suggesting that the host’s response/s to CoV might play role/s in determining the form of CoV disease [163]. PEHV replicates primarily in the respiratory tract and can further spread to the central nervous system through the peripheral nerves, resulting in encephalomyelitis, vomiting (due to infection of vagal ganglia), and/or wasting (due to lesions in the myenteric plexus) in piglets [37].

## 7. Therapeutics against SARS-CoV-2 and the Animal Coronaviruses

Since the various therapeutic strategies against SARS-CoV-2 have been extensively reviewed elsewhere [164,165], this section will primarily focus on similar therapeutic approaches against animal CoVs. Among the antivirals, Remdesivir (GS-5734), an adenosine nucleotide analogue prodrug that inhibits the viral RNA-dependent RNA polymerase (RdRp), has been considered as one of the most promising drugs against SARS-CoV-2 [164,165]. In vitro studies have revealed that remdesivir is highly efficacious against PDCoV [166]. On the other hand, mutation studies with MHV have shown that viruses with improved proofreading activity exhibit resistance, whilst those devoid of proofreading ability were more sensitive to remdesivir [167,168]. The parent nucleoside of remdesivir, GS-441524, was shown to be a safe and promising therapeutic candidate against FIPV in tissue culture, experimental, and natural cat infection studies [168,169,170,171]). Because of its synthetic simplicity and in vivo efficacy under veterinary settings, GS-441524 has been proposed to be more superior to remdesivir for COVID-19 treatment [172].

Although randomized clinical trials with drugs Lopinavir/Ritonavir revealed little/no benefit in SARS-CoV-2 patients [164,165,173], the antiviral activities of protease inhibitors have been evaluated against CoVs in different animal species. The 3C-like protease inhibitor (3CLpro) GC376, previously shown to block FIPV infection in naturally and experimentally infected cats, was found to inhibit replication of SARS-CoV-2 and PEDV in cell culture [174,175,176,177]. By FRET assays, broadly acting 3CLpro inhibitors have been identified against feline, ferret, and mink CoVs [178]. In another study, Galanthus nivalis agglutinin and nelfinavir were shown to synergistically exert an antiviral effect on FCoVs in vitro [179]. The chemotherapeutic drug, 6-thioguanine, was found to noncompetitively inhibit the PEDV papain-like protease 2 [180]. Interestingly, 3CLpro inhibitor resistant MHV mutants were shown to be attenuated for replication and pathogenesis in mice, revealing a low genetic barrier but high fitness cost of resistance for CoVs [181].

The efficacy of ribavirin (a synthetic guanosine nucleoside analogue that blocks viral RNA synthesis and viral mRNA capping) against SARS-CoVs has been questioned [164], although promising results were obtained when ribavirin was used in combination with protease inhibitors and interferon-ß-1b (IFN-ß-1b) during a randomized clinical trial against SARS-CoV-2 [182]. A combination of ribavirin and recombinant human leukocyte (alpha) IFN (rHuIFN-α) caused a significant increase in antiviral activities against FIPV compared with that produced by ribavirin, or rHuIFN-α alone [183]. However, ribavirin is not recommended for treatment of FIPV because of its intrinsic toxicity, marginal antiviral activities (in vivo) at maximal doses, and low therapeutic index against FIPV [184]. Ribovirin was shown to effectively inhibit PEDV infection in cell culture at subcytotoxic doses [185].

Chloroquine Phosphate (CQ) and Hydroxychloroquine (HCQ), approved by the FDA for the treatment of malaria, lupus erythematosus, and extraintestinal amebiasis, have received a lot of attention in the treatment of SARS-CoV-2 [164]. Both forms are believed to exhibit antiviral activity by increasing endosomal pH (thereby, impairing fusion between the virus envelope and host cell endosomal membrane), interfering with the glycosylation of ACE2, and exerting immunomodulatory effects [164,186]. The therapeutic effect of CQ against FIPV was investigated in vivo and in vitro by Takano et al. (2013) [187]. Although CQ was found to exhibit an inhibitory effect against the replication of FIPV, and demonstrated anti-inflammatory effects (in vitro), it did not induce any antiviral effects in cells already infected with the virus. In another study, a combination of HCQ and recombinant feline IFN-ω (rfIFN-ω) resulted in increased antiviral activity against type I FIPV infection [188]. HCQ in combination with homoharringtonine (HHT) was found to exhibit higher antiviral activity against PEDV than that with either agent alone [189]. On the other hand, HCQ did not show antiviral activity or clinical efficacy in macaques infected with SARS-CoV-2 [190].

Ivermectin, a veterinary drug, has been shown to possess antimicrobial, antiviral, and anticancer properties, and is being evaluated as a potential candidate in the treatment of SARS-CoV-2 [191,192]. The antiviral effect of ivermectin might be due to inhibition of the importin (IMP) a/ß receptor, which is responsible for the nuclear transport of viral proteins [193]. Although there is no information on the antiviral activity of ivermectin against animal CoVs so far, the antiviral effects of ivermectin have been observed against various RNA (avian influenza A virus, Newcastle virus, porcine reproductive and respiratory syndrome virus, Venezuelan equine encephalitis virus, West Nile virus) and DNA (bovine herpesvirus 1, equine herpes type 1, porcine circovirus 2, and pseudorabies) viruses of animals [192].

Cyclophilin inhibitors (Cyclosporine A and analogues such as Alisporivir) are immunosuppressive drugs with anti-calcineurin properties [194]. Alisporivir has been shown to exhibit strong, dose-dependent antiviral activity against SARS-CoV-2 in vitro, and is being considered for a phase-2, proof-of-concept clinical trial [195,196]. However, concerns have been raised about the safety of cyclosporine in SARS-CoV-2 patients [194]. Previously, cyclophilin inhibitors have been shown to inhibit the replication of animal CoVs (FCoV, IBV, and MHV) and human CoVs (HCoV-229E, HCoV-NL63, MERS-CoV, and SARS-CoV) [194,195,197].

Antiviral peptides (AVPs) are short and simple amino acid sequences that can exert antiviral effects by targeting different viral components [198]. Synthetic peptides targeting the viral envelope (Mucroporin-M1) or S protein (EK1 and EK1C4), or AVPs inhibiting the late endosomal acidification in infected host cells, thereby preventing the release of viral RNA (P9 peptide), have been found to exhibit anti-CoV activities [198]. Lipid conjugation to an inhibitory peptide derived from the C-terminal heptad repeat (HRC) domain of SARS-CoV-2 S protein was found to inhibit virus infection in Vero E6 cells, and block the spread of SARS-CoV-2 in human airway epithelial (HAE) cultures [199]. Neutralization of the polybasic cleavage site of the SARS-CoV-2 S protein with a negatively charged tetrapeptide, GluGluLeuGlu, was shown to lessen the RBD-ACE2 binding strength by 34% [144]. The antiviral effects of several peptides targeting the S1 domain of FIPV S protein have also been studied, revealing inhibitory effects on virus infection [200]. In another study, combination of a synthetic peptide FP5 (designed from the putative HR2 domain of the spike protein of FCoV) with interferon-alpha (IFN-α) was found to significantly inhibit FCoV replication in cultured *Felis catus* whole fetus-4 (fcwf-4) cells [201]. Peptides derived from HR2 were shown to inhibit PEDV entry and infection in cell culture [202]. In addition to antiviral effects, AVPs can be used as immunomodulators, or shield the host receptors from viral recognition and attachment [198]. Human Intestinal Defensin-5 was shown to cloak several sites in the ligand binding domain of ACE2, which are crucial for effective binding of the S protein of SARS-CoV-2 to ACE2 [203].

Lipid metabolism plays a crucial role in the viral replication cycle, and virus infection-associated host lipid metabolic remodeling has been demonstrated for CoVs [204,205,206]. Therefore, drugs targeting the lipid metabolic pathways that are pivotal to CoV infection could be explored as potential therapeutic options against SARS-CoV-2. In a recent study, cholesterol 25-hydroxylase was shown to inhibit SARS-CoV-2 and other CoVs by depleting accessible cholesterol from the plasma membrane, thereby suppressing virus–host cell membrane fusion [207]. Agents that deplete/inhibit cholesterol synthesis/transport have been found to inhibit the replication of animal CoVs in vitro, such as U18666A-inhibited FIPV and methyl-beta-cyclodextrin-inhibited FIPV and PEDV [208,209]. Linoleic acid (LA) is a free fatty acid with anti-inflammatory and immunomodulatory properties [205]. Exogenous supplementation of linoleic acid (LA) has been shown to inhibit virus replication in HCoV-229E- or MERS-CoV-infected cells [205]. Recently, analysis of the 2.85 Å cryo-electron microscopy structure of the SARS-CoV-2 S protein revealed three composite binding pockets that tightly bind LA [210]. It has been hypothesized that sequestration of LA by SARS-CoV-2 might enhance CoV-mediated immune dysregulation and inflammation [210]. On the other hand, binding of LA to these pockets generated a locked S protein structure, which reduced virus RBD-ACE2 interaction in vitro, offering the prospects of developing intervention strategies that target the LA binding sites of SARS-CoV-2 [210].

Neutralizing monoclonal antibodies (mAbs) that target the RBD on the S protein of SARS-CoV-2 have shown the potential for both therapeutic and prophylactic applications, and several SARS-CoV-2 mAbs are currently poised to enter into human clinical trials [211,212,213]. Neutralizing antibodies targeting the S protein have been studied in various animal CoVs: PEDV and TGEV [214,215,216,217,218], BCoV [219] and IBV [220,221]. Monoclonal antibodies targeting other CoV proteins have also been investigated, such as four of the six mAbs against BCoV E2 and E3 glycoproteins exhibited neutralization activity in vivo [222]. However, none of these mAbs have been used in routine treatment protocols. Monoclonal antibodies attenuating the cytokine-mediated inflammatory response, such as tocilizumab, a humanized mAb targeting IL-6 receptor, have been used in the treatment of SARs-CoV-2 patients [165]. Monoclonal antibodies against tumor necrosis factor-alpha (TNF-α, involved in aggravating pathology of FIPV) were shown to be effective in preventing progression to FIP in experimentally infected cats [223].

Short interference RNAs (siRNAs) are small non-coding double-stranded RNA molecules that control gene expression by mediating the degradation of target RNA in a sequence-specific manner [224]. siRNAs have been used in the treatment of various virus infections [224,225,226]. siRNAs inhibited SARS-CoV infection and replication in vivo, and suppressed SARS-like symptoms in nonhuman primates [226]. A panel of eight synthetic siRNAs targeting four different regions of the FCoV genome were found to inhibit virus replication in vitro [227]. In another study, different combinations of five siRNAs targeting coding and noncoding regions of the FCoV genome achieved more effective viral inhibition compared to individual siRNAs [228]. Combined siRNA therapy was shown to prevent escape of siRNA-resistant FCoV mutants in vitro [229]. siRNAs restricted viral protein expression and inhibited the replication of PEDV, PDCoV, PHEV, and SADS-CoV in vitro [230,231,232]. siRNA expression inhibited virus replication, lowered inflammation, significantly decreased mortality, and improved weight gain in poultry experimentally infected with IBV [233].

Interferons (IFNs) are key components of the innate immune responses against viruses [225,226]. IFN-ß has been used in combination with other drugs in clinical trials against SARS-CoV-2 [182]. Although recombinant feline interferon-omega (rFeIFN-ω) improved clinical signs and reduced concurrent viral excretion in cats naturally infected with retroviruses, it did not prolong survival time or improve quality of life in FIPV-positive cats [234,235]. Recombinant feline interferon (rFeIFN) KT-80 exerted inhibitory effects on the replication of FCoV and other enteric viruses in vitro [236]. Porcine IFN-λ3 and IFN-γ demonstrated antiviral activity against PEDV and TGEV, respectively, in vitro [237,238]. Recombinant chicken IFN type I (rChIFN-α) inhibited the replication of IBV in vitro, and was shown to protect chicks by delaying the onset of disease and reducing the severity of illness [239].

Dexamethasone, a corticosteroid with anti-inflammatory and immunosuppressant effects, has been shown to improve survival and mortality in patients critically ill with SARS-CoV-2 [164]. In experimental studies, dexamethasone treatment alleviated early proinflammatory responses in PRCoV infected pigs; however, continued use for a longer period enhanced viral replication and exacerbated infection by decreasing cellular immune responses [240,241]. In another study, experimental challenge with TGEV induced profuse diarrhea in dexamethasone-treated pigs [242]. In cattle, dexamethasone was shown to cause immune suppression and influence the occurrence of diarrhea and virus shedding in calves challenged with BCoV [243].

Other therapeutic considerations against SARS-CoV-2 that have been tried against animal CoVs include (i) glycopeptide antibiotics and pyridine N-oxide derivatives (inhibited FIPV and SARS-CoV in vitro) [244,245]; (ii) lithium (demonstrated antiviral effect against PEDV in vitro) [246]; (iii) antifungal drug itraconazole (antiviral activity against FIPV) [247]; (iv) Janus-Kinase inhibitors (inhibited replication of TGEV) [248]; (v) immunostimulants (shown to increase survival time in cats with dry form of FIPV) [249]; (vi) stem cells (transduction of hematopoietic stem cells stimulated RNA interference against FIPV) [250]; and (vii) natural product-derived phytochemicals [251].

To date, there are no approved treatment protocols against the animal CoVs [252]. On the other hand, a recent study conducted by the World Health Organization Solidarity trial consortium at 405 hospitals in 30 countries revealed that remdesivir, hydroxychloroquine, lopinavir, and IFN regimens may have little or no effect on hospitalized cases of SARS-CoV-2, as evident from the data on overall mortality, initiation of ventilation, and duration of hospital stay [253].

## 8. Prophylaxis against SARS-CoV-2 and the Animal Coronaviruses

Prophylactic strategies against SARS-CoV-2 have been comprehensively reviewed by Jeyanathan et al. (2020) [10] and several others. At least 166 vaccine candidates, based on different platforms (DNA-based, inactivated virus, live attenuated virus, mRNA-based, protein subunit, virus-vectored (replicating and non-replicating), virus-like particles), are in various stages of preclinical, or clinical development. Whilst the race for developing a vaccine against human CoVs has gained momentum, licensed CoV vaccines are already available for use in livestock (cattle, pigs, and poultry) and companion (cats and dogs) animals [30]. Some of the licensed animal CoV vaccines include candidates against other pathogens, such as BCoV with rotavirus, clostridia and *Escherichia coli*, CCoV with canine adenovirus, canine distemper virus, canine parvovirus-2, Leptospira and Lyme disease, PEDV with rotavirus and TGEV, and IBV with Newcastle disease, or infectious bursal disease and reovirus [30,32,254]. The different prophylactic strategies against animal CoVs are shown in Table 1. Although the currently licensed animal CoV vaccines appeared to reduce mortality/viral shedding, none seem to offer complete protection [33]. Other concerns associated with the animal CoV vaccines included a relatively short duration of protective immunity, lack of a clear understanding of the correlates of protection against CoVs, emergence of multiple serotypes/subtypes that influence vaccine efficacy, and/or ADE enhanced disease [14,17,18,22,30,32,33,254,255].

One of the potential safety issues with SARS-CoV-2 vaccines is possible disease enhancement by ADE [256]. Although antibodies generally play protective roles, non/sub-neutralizing antibodies might promote enhanced virus uptake into host cells via Fcγ receptors (FcγR), aggravating the disease condition. This phenomenon, known as ADE, has been observed with dengue in humans, and typically involves macrophages [256,257]. There is no direct evidence for ADE in SARS-CoV-2 infection so far, and macrophages/monocytes do not appear to be the primary targets of the virus [10]. However, FcγRs have been proposed to facilitate SARS-CoV uptake into monocytes/macrophages and B cells through pre-existing virus-specific antibodies, and ADE has been reported in some laboratory animals immunized with experimental SARS-CoV vaccines [10,258,259,260]. Furthermore, exaggerated cytokine profiles, resembling those of macrophage activation syndrome, have been observed in patients infected with SARS-CoV-2 [9,137]. ADE-enhanced FIPV infection has been demonstrated in experimentally infected cats, and was best observed after re-infection with the same serotype [17]. The antibody-opsonized FIPV virions interact with the FcγRs on macrophages/monocytes, and are eventually internalized and released into the cytoplasm of the host cell [16]. In experimental challenge studies, conventional domestic cats immunized with recombinant FIPV vaccine, or antibodies against FIPV spike proteins developed an accelerated disease course, whilst specific pathogen-free cats were found to be protected against FIPV [17,261,262,263]. In a recent study, 50% of passively immunized cats that were inoculated orally with FIPV-I KU-2 developed FIP through ADE [264]. ADE-enhanced FIPV is believed to be of low relevance under natural settings [103]. Although the only licensed intranasal FIP vaccine was shown to be safe in cats over 16 weeks of age, it is not recommended by the American Association of Feline Practitioners because of conflicting results between efficacy studies, and that most kittens are endemic to FCoV before 16 weeks of age [41].

There is no evidence for antigenic drift or antigenic shift among SARS-CoV-2 strains circulating in humans so far [81]. However, the possibility of such events cannot be ruled out, especially with the extended human-to-human transmission, long duration of the pandemic, and possible transmission to other hosts [7,11,81,129]. Both antigenic drift and shift have been shown to influence vaccine efficacy against animal CoVs [18,19,21,22,32,254]. The IBV genome, especially the antigenically significant S protein encoding gene, evolves rapidly by random mutation (antigenic drift) and/or recombination (antigenic shift) events, resulting in a wide variety of genotypes, serotypes, and pathotypes [21,22]. Recombination events have not only been reported between field strains, but also between live attenuated vaccine and field strains. Mutations inducing even minor changes (<5%) in the S1 protein sequence might influence IBV vaccine efficacy [30]. Since the different serotypes of IBV do not exhibit cross-protection, vaccination programs against IBV adopt either a “multi-monovalent”, or “protectotype” strategy [254]. Because of high strain diversity between countries/regions, the attenuated viruses used in the current IBV vaccines vary depending on the geographical location (M41 (Massachusetts), Arkansas and Connecticut in North America, 4/91 and D274 in Europe, and QX in China). Similar to IBV, recombination events including a recent report on recombination between a highly pathogenic field PEDV strain and a low pathogenic attenuated vaccine PEDV strain have been found to negatively impact the efficacy of the current PEDV vaccines [18,32,255]. On the other hand, genetic diversity in the CoV genome has also resulted in the emergence of naturally attenuated viruses that offer protection against the virulent counterparts, such as PRCoV (deletion mutant of TGEV, causes mild respiratory disease), which has been attributed to the worldwide reduction in outbreaks of severe gastroenteritis with TGEV [20,153].

The route of vaccination would be crucial in determining vaccine efficacy against SARS-CoV-2, as intranasal vaccines elicit a strong local IgA response and fewer systemic antibodies, whilst parenteral vaccines induce sufficient virus neutralizing antibodies in serum that also appear in respiratory mucosa, but do not elicit an effective IgA response [10]. As with SARS-CoV-2, the correlates of immunity to respiratory CoV infections in animals remain to be clearly elucidated [20,30,33]. In poultry, the primary response to an ocular IBV vaccine (attenuated Ark DPI vaccine) was dominated by IBV-specific IgA, whilst the secondary response to the vaccine was characterized by an increase in IgG antibody titers and a decrease in IgA antibody titers in both tears and plasma [265]. High titers of viral neutralizing antibodies in serum have been proposed to prevent the spread of IBV from the respiratory tract to other organs [33]. Serum neutralizing antibodies were correlated with the protection of naturally infected feedlot cattle against bovine respiratory disease complex (BDRC), whilst intranasal vaccination of calves with a live attenuated BCoV vaccine (licensed for oral use against BCoV-induced diarrhea) prior to entering feedlot minimized the risk of BDRC in calves [20]. With swine enteric CoVs, IgA in milk and colostrum were found to be correlates of passive immunity in piglets [32]. In general, live attenuated CoV vaccines were shown to be more effective than inactivated CoV vaccines in animals, although the former carries the risk of reversal to virulence [21,22,30,32,254].

In conclusion, it should be noted that the veterinary CoV vaccines are unlikely to prevent COVID-19 in humans, as the animal vaccine candidates are antigenically unrelated to SARS-CoV-2. Furthermore, the licensed veterinary CoV vaccines are directed against diseases of animals that do not exactly resemble the pathology (severe atypical pneumonia) seen with SARS-CoV-2 [14,17,19,20,22]. Nevertheless, information on various aspects of animal CoV vaccines (development, efficacy, and setbacks) might prove useful in the development of an effective and safe vaccine against SARS-CoV-2.

## 9. Conclusions

Satirically, the crown-like morphology of SARS-CoV-2 indeed befits the virus, for SARS-CoV-2 would surely be crowned as one of the most devastating viruses of recent times with regards to its impact on health, economies, and societies worldwide [2,3,5,6]. Although the current SARS-CoV-2 pandemic has triggered a global wave of research studies on CoVs at an unprecedented scale, human CoVs attracted attention only after the SARS outbreak of 2002–2003. On the other hand, animal CoVs have long been identified as major causes of mortality and morbidity in livestock and companion animals. As a result, animal CoVs have been studied extensively, yielding a plethora of information on CoV evolution, transmission including interspecies transmission events, pathogenesis including immunopathology, therapeutics, and prophylaxis, evaluation of which might allow for a better understanding of CoV disease in humans.

Surveillance and genome sequencing of CoVs from various animals species, including wildlife, were extremely crucial in tracing the possible origin and transmission of SARS-CoV-2 (and that of SARS-CoV and MERS), suggesting that these viruses were most likely derived from bats with/without an animal intermediate host (Figure 3) [11,24,139]. Antibodies against SARS-CoV have been detected in humans living near bat caves in China [89]. Recently, SARS-COV-2 has been reported in cats, dogs, minks, and captive wildlife, indicating possible human-to-animal transmission events [129]. Taken together, these observations underscored the significance of continuous monitoring of zoonotic and zooanthroponotic pathogens in animals, especially wildlife, and in humans residing near wildlife habitats. Once again, evolutionary analysis of SARS-CoV-2 reiterated the importance of reducing contact potential between humans and wildlife by addressing climate change and deforestation, and banning wildlife trade/wet animal markets [11,266,267].

Compared to the other human CoVs and many other zoonotic viruses, the rapidity and extent of human-to-human spread of SARS-CoV-2 has intrigued many researchers [1,3,7]. Although remarkable, this observation might not appear surprising, at least to animal virologists, for studies on animal CoVs have previously revealed the capability of CoVs to jump the species barrier, and successfully adapt and spread in a new host species. Widely prevalent animal CoVs, such as FCoV-II, PEDV, and TGEV, have been proposed to be derived from heterologous hosts including unrelated animal species (Figure 3) [13,15,16,17,18,19].

Studies on animal CoVs have revealed the enigmatic nature of CoVs, such as alterations in tissue tropism (pantropic CCoV-IIa, FIPV, and PRCoV), multi-organ tropism (BCoV, pantropic CCoV-IIa, FIPV, IBV, MHV, and PHEV), and/or immunopathology (FIPV) [14,16,17,18,19,20,21,22,23,37]. Although changes in the CoV genome have been proposed to induce alterations in tissue tropism, except for that of PRCoV, the molecular basis for such events remain to be clearly elucidated. Interestingly, SARS-CoV-2, considered as a respiratory pathogen, has been associated with extra-clinical manifestations, and viral RNA has been detected in various organs and fecal samples [8,9,137]. Furthermore, the cytokine storm observed in SARS-CoV-2 patients mirrors that in cats with FIP [9,17] (Figure 4). Considering the above, it would be interesting to compare the genetic variations and pathogenesis of SARS-CoV-2 strains from various organs.

The development of an effective and safe vaccine against SARS-CoV-2 is of paramount importance. Although there are no licensed vaccines against CoVs in humans so far, the various issues (lack of complete protection, short duration of immunity, correlates of protection against CoVs, emergence of multiple serotypes/subtypes including virulent recombinants between vaccine and field strains, and/or ADE enhanced disease) observed with the veterinary CoV vaccines should be taken into consideration while devising immunization strategies against SARS-CoV-2 [17,18,22,30,32,33,254,255].

SARS-CoV-2 has challenged the very basic tenet of human existence: “United We Stand, Divided We Fall”. Measures aimed at reducing human-to-human transmission have resulted in a crisis situation, where, unfortunately, “Divided We Stand, United We Fall” has become the motto for survival. However, pandemics, especially those caused by zoonotic pathogens such as SARS-CoV-2, could be avoided, or effectively controlled, by adopting a One Health approach that unites the various streams of animal, human, and environmental health, thereby reinforcing the concept of “United We Stand, Divided We Fall”.

## Figures and Tables

**Table 1:** Comparisons of the various features of SARS-CoV-2 with those of coronaviruses (CoVs) in livestock and companion animals.

Host	Common Name of Virus, *Subgenus*, *Genus*	Receptor/s	Clinical Manifestation/s	Vaccine Strategies	References
Humans	SARS-CoV-2, *Sarbecovirus, Betacoronavirus*	ACE2	Fever, cough, atypical pneumonia, acute respiratory distress syndrome, diarrhea, and multiple organ failure	Viral-vectored; mRNA-based; DNA-based; live attenuated; inactivated, protein subunit; virus-like particle	[9,10,29]
Cattle	Bovine coronavirus (BCoV), *Embecovirus*, *Betacoronavirus*	Neu5,9Ac2/ HLA-I	Diarrhea, winter dysentery, and/or respiratory disease	Live attenuated; inactivated	[20,29,30,31]
Pigs	Transmissible gastroenteritis virus (TGEV), *Tegacovirus, Alphacoronavirus*	APN	Diarrhea, vomiting	Live attenuated; inactivated; recombinant proteins expressed in baculovirus, yeast, and plants; DNA-based	[18,19,20,29,30,32]
	Porcine respiratory coronavirus (PRCoV), *Tegacovirus, Alphacoronavirus*	APN	Fever, atypical pneumonia	Recombinant adenovirus vector-based	[19,20,29,33]
	Porcine epidemic diarrhea virus (PEDV), *Pedacovirus, Alphacoronavirus*	Sialic acid? APN ^1^	Diarrhea, vomiting	Live attenuated; inactivated; recombinant proteins expressed in baculovirus, yeast, plants and bacteria; DNA-based	[18,19,29,30,32]
	Swine acute diarrhea syndrome coronavirus (SADS-CoV)/Swine enteric alphacoronavirus (SeACoV), *Rhinacovirus, Alphacoronavirus*	Not determined	Diarrhea, vomiting	Inactivated (reported as unpublished data in Zhou et al. (2019) [34])	[18,29,34,35]
	Porcine deltacoronavirus (PDCoV), *Buldecovirus, Deltacoronavirus*	APN, other unknown receptor/s?	Diarrhea, vomiting	Inactivated	[18,29,36]
	Porcine hemagglutinating encephalomyelitis virus (PHEV), *Embecovirus, Betacoronavirus*	Neu5,9Ac2	Neurological signs, vomiting, wasting	Inactivated and DNA-based vaccine evaluated in mice	[29,37,38]
Poultry	Infectious bronchitis virus (IBV) *Igacovirus, Gammacoronavirus*	Neu5Gc	Respiratory disease, renal injury, and/or reduction in egg quality and quantity	Live attenuated; inactivated; viral vector-based; subunit/peptide-based; plasmid DNA-based	[21,22,29,39,40]
Cats	Feline coronavirus I (FCoV-I), Feline coronavirus II (FCoV-II), *Tegacovirus, Alphacoronavirus*	APN	Mild diarrhea, asymptomatic infection (feline enteric CoV (FECoV)). Body cavity effusions and fibrinous and/or granulomatous inflammatory lesions in various organs (seen in wet form of feline infectious peritonitis virus (FIPV)). Uveitis and neurological signs (common in dry form of FIPV).	Modified live intranasal vaccine (was licensed, but not recommended by American Association of Feline Practitioners).	[17,29,41]
Dogs	Canine coronavirus I (CCoV-I), Canine coronavirus II (CCoV-II), *Tegacovirus, Alphacoronavirus*	APN	Mild diarrhea. Systemic disease with vomiting, hemorrhagic diarrhea, and neurologic signs (pantropic CCoV-II)	Modified live; inactivated	[14,29,30]
	Canine respiratory coronavirus (CRCoV), *Embecovirus, Betacoronavirus*	HLA-I	Mild respiratory disease	No information available	[14,29,42]

^1^ It has been demonstrated that APN is not a functional receptor of PEDV [18].
